# The circle to lariat ratio of the Ll.LtrB group II intron from *Lactococcus lactis* is greatly influenced by a variety of biological determinants *in vivo*

**DOI:** 10.1371/journal.pone.0237367

**Published:** 2020-08-18

**Authors:** Caroline Monat, Benoit Cousineau

**Affiliations:** Department of Microbiology and Immunology, McGill University, Montréal, Québec, Canada; Iowa State University, UNITED STATES

## Abstract

Bacterial group II introns mostly behave as versatile retromobile genetic elements going through distinct cycles of gain and loss. These large RNA molecules are also ribozymes splicing autocatalytically from their interrupted pre-mRNA transcripts by two different concurrent pathways, branching and circularization. These two splicing pathways were shown to release in bacterial cells significant amounts of branched intron lariats and perfect end-to-end intron circles respectively. On one hand, released intron lariats can invade new sites in RNA and/or DNA by reverse branching while released intron circles are dead end spliced products since they cannot reverse splice through circularization. The presence of two parallel and competing group II intron splicing pathways in bacteria led us to investigate the conditions that influence the overall circle to lariat ratio *in vivo*. Here we unveil that removing a prominent processing site within the Ll.LtrB group II intron, raising growth temperature of *Lactococcus lactis* host cells and increasing the expression level of the intron-interrupted gene all increased the relative amount of released intron circles compared to lariats. Strengthening and weakening the base pairing interaction between the intron and its upstream exon respectively increased and decreased the overall levels of released intron circles in comparison to lariats. Host environment was also found to impact the circle to lariat ratio of the Ll.LtrB and Ll.RlxA group II introns from *L*. *lactis* and the Ef.PcfG intron from *Enterococcus faecalis*. Overall, our data show that multiple factors significantly influence the balance between released intron circles and lariats in bacterial cells.

## Introduction

**Fig 1:**
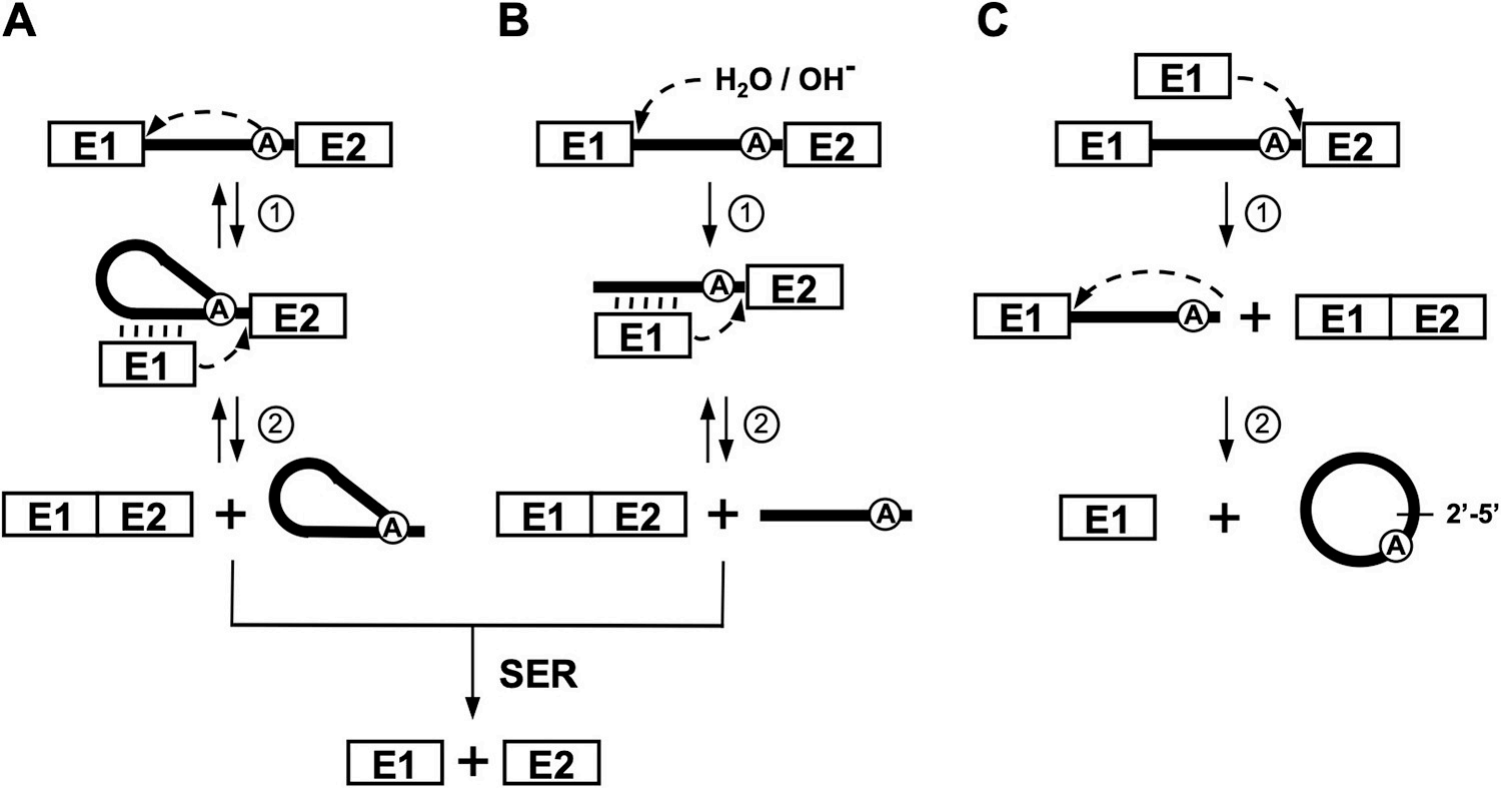
Group II intron splicing pathways in bacterial cells. (A) Branching pathway. Following transcription of the intron-interrupted gene, the 2´-OH residue of the branch-point A nucleotide initiates the first nucleophilic attack at the E1-intron 5′ splice junction (step 1). This transesterification reaction connects the 5´ end of the intron to the 2´-OH residue of the branch point A and releases E1 that remains associated to the intron through EBS1/2-IBS1/2 base pairing interactions (vertical lines). The liberated 3´-OH at the end of E1 then initiates the second nucleophilic attack at the intron-E2 3´ splice junction, ligating the two exons (E1-E2) and releasing the intron as a lariat or branched structure (step 2). (B) Hydrolytic pathway. The first transesterification reaction at the 5′ splice junction is initiated by an external nucleophile (H_2_O / OH^-^) leading to the release of E1 (step 1). The liberated 3´-OH at the end of E1 then initiates the second nucleophilic attack at the 3´ splice junction releasing the intron in linear form while ligating the two exons (E1-E2) (step 2). (C) Circularization pathway. The first nucleophilic attack takes place at the intron-E2 3´ splice junction and is initiated by the 3´-OH of a free E1 (step 1), generating ligated exons (E1-E2) and a circularization splicing intermediate where the 5′ end of the linear intron is still attached to E1. This reaction is not always accurate generating a series of circularization intermediates where the first nucleotides (nts) of E2 are still attached to the intron 3′ end [4]. Next, the 2´-OH of the last intron residue initiates the second nucleophilic reaction at the E1-intron 5´ splice junction resulting in the release of perfect head to tail intron circles and free E1 (step 2). A potential source of free E1 is the spliced exon reopening reaction (panel A and B, SER) where linear introns and lariats can recognize and hydrolyze ligated exons (E1-E2) at the splice junction.

Group II introns are large ribozymes that share a complex but universally conserved RNA secondary structure composed of six domains (DI-DVI) linked together by a central core [1, 2]. Some of these RNA enzymes harbor an open reading frame in DIV coding for a multi-functional intron-encoded protein (IEP) with reverse transcriptase, maturase and sometimes endonuclease activity. The maturase function of IEPs is essential for the accurate folding and subsequent splicing of group II introns *in vivo* [1, 2]. After binding to their IEP and adopting an active tridimensional structure, these ribozymes excise autocatalytically from their primary RNA transcripts by two consecutive transesterification reactions while concurrently ligating their flanking exons (Fig 1, steps 1 and 2) [3]. Group II introns can self-splice by three different splicing pathways, branching (Fig 1A), hydrolysis (Fig 1B) and circularization (Fig 1C) and be released respectively as branched structures called lariats, linear introns and perfect end-to-end closed circles [3–6].

Ancestral group II introns are regarded as important retromobile elements that extensively shaped the origin as well as the evolution of contemporary eukaryotic genomes [7]. They are the proposed progenitors of the highly abundant non-long terminal repeats (non-LTR) retroelements and nuclear spliceosomal introns. Moreover, the spliceosome machinery and telomerase enzyme are also considered evolutionarily derived from group II introns. Overall, the putative group II intron derivatives constitute more than half of the human genome and significantly contribute to key functions in eukaryotes [2, 7–10]. One of these important functions is the generation of genetic diversity. For example, spliceosomal introns generate significant genetic diversity at the RNA level through alternative splicing whereas the mobility of non-LTR retroelements within genomes produces genetic diversity at the DNA level [11, 12].

Contemporary group II introns have a broad phylogenetic distribution that is rather irregular [1, 2, 13]. They are found in one quarter of all sequenced bacterial genomes, within several plasmids and in some bacterial-derived organelles including mitochondria and chloroplasts in plants as well as fungal mitochondria. On the other hand, only few examples were yet unveiled in archaea while no active copies are present in the nuclear genome of eukaryotes where they appear to be functionally excluded [8]. Organellar group II introns, in both mitochondrial and chloroplastic genomes, generally interrupt housekeeping genes and almost solely act as ribozymes only performing basic splicing functions. Conversely, bacterial group II introns are predominantly found within other mobile genetic elements or in non-coding sequences and behave as efficient mobile retroelements going through cycles of gain and loss [2, 13–15]. Altogether, these observations have led to a general consensus that bacterial group II introns behave solely as selfish retromobile elements, conferring no beneficial function to their hosts [13]. However, a potentially beneficial function for group II introns has recently been observed, which combines aspects of both branching and circularization [6]. Ll.LtrB, the model group II intron from the gram-positive bacterium *Lactococcus lactis*, was shown to generate genetic diversity at the RNA level by shuffling mRNA fragments. First, released intron lariats reverse the branching pathway to generate a population of intron-invaded mRNA transcripts *in vivo*. Next, they self-splice through circularization, joining non-contiguous mRNA fragments by *trans*-splicing [6]. This delicate balance between branching and circularization has the effect of shuffling fragments of interrupted transcripts together and increasing the genetic diversity of the bacterial transcriptome by producing chimeric mRNA transcripts [6].

Although branching is the primary and most studied group II intron splicing pathway, circularization was always considered as a secondary pathway, principally studied *in vitro* and not as properly characterized [3, 16]. We recently conducted the first molecular analyses of the group II intron circularization pathway [4–6]. Our work demonstrated that a significant proportion of Ll.LtrB, excises through the circularization pathway while no trace of perfect linear intron could be detected [4, 5]. Importantly, we detected the presence of perfect intron circles for a series of Ll.LtrB variants and demonstrated that both branching and circularization take place concurrently *in vivo*, showing that circularization is a significant and conserved splicing pathway for bacterial group II introns. Moreover, the accumulation of substantial amounts of intron RNA circles in bacterial cells suggests that they may play a role in providing useful function(s) to their hosts [4, 5]. The coexistence of two competing splicing pathways and their interplay during *trans*-splicing raises the question of which factors influence the balance between circularization and branching and ultimately the overall circle to lariat ratio *in vivo* [4–6].

Here we show that several factors including intron processing, growth phase and temperature, strength of exon 1 recognition, expression level as well as the host cellular environment greatly influence the overall circle to lariat ratio of bacterial group II introns *in vivo*. Taken together, our findings corroborate the previously proposed circularization pathway and confirm the existence of two significant concurrent splicing pathways *in vivo*, branching and circularization. It also unveils multiple factors that control the balance between released group II intron circles and lariats in bacterial cells.

## Materials and methods

### Bacterial strains and plasmids

*Lactococcus lactis* strains (NZ9800, NZ9800Δ*ltrB* (Tet^R^) [17], LMG9447, NZ3900) were grown in M17 media supplemented with 0.5% glucose (GM17) at 30°C without shaking. The *Enterococcus faecalis* strains (SF24397 [18], JH2-2) were grown in BHI at 37°C without shaking. The *Escherichia coli* strain DH10β, used for cloning purposes, was grown in LB at 37°C with shaking. Antibiotics were used at the following concentrations: chloramphenicol (Cam^R^), 10 μg/ml; spectinomycin (Spc^R^), 300 μg/ml; erythromycin (Erm^R^), 300 μg/ml.

Some plasmids used in this study were previously described: pDL-P_23_^2^-Ll.LtrB-WT [19], pDL-P_23_^2^-Ll.LtrB-ΔA [5], pLE-P_nis_-Ll.LtrB-WT [14], pLE-P_nis_-Ef.PcfG-WT [14]. The intron-interrupted relaxase gene *rlxA* from LMG9447 was amplified by PCR and cloned downstream of the P_23_ constitutive promoter in pDL-P_23_^2^ (pDL-P_23_^2^-Ll.RlxA-WT) and pLKV1-P_23_^2^ (pLKV1-P_23_^2^-Ll.RlxA-WT), and downstream of the nisin-inducible promoter in pLE-P_nis_ (pLE-P_nis_-Ll.RlxA-WT). The intron-interrupted relaxase gene *pcfG* from SF24397 was amplified by PCR and cloned downstream of the P_23_ constitutive promoter in pDL-P_23_^2^ (pDL-P_23_^2^-Ef.PcfG-WT) and pLKV1 (pLKV1-P_23_^2^-Ef.PcfG-WT). The intron-interrupted relaxase gene *ltrB* from NZ9800 and its branch-point mutant were amplified by PCR and cloned downstream of the P_23_ constitutive promoter in pLKV1 (pLKV1-P_23_^2^Ll.LtrB-WT and pLKV1-P_23_^2^-Ll.LtrB-ΔA). The following intron variants were obtained by site-directed mutagenesis (New England Biolabs^®^ Q5^®^ Site-Directed-Mutagenesis Kit): pDL-P_23_^2^-Ll.LtrB-Mut-138, pDL-P_23_^2^-Ll.LtrB-GC-rich, pDL-P_23_^2^-Ll.LtrB-AU-rich, pDL-P_23_^2^-Ll.LtrB-Mut-IBS2, pDL-P_23_^2^-Ll.LtrB-IBS2-EBS2 swap, pDL-P_23_^2^-Ll.LtrB-Mut-EBS1, pDL-P_23_^2^-Ll.LtrB-IBS1-EBS1 swap. Primers used for cloning and site-directed mutagenesis are shown in Table 1.

**Table 1:** Primers.

Primer location	Function	Sequence (5′-3′)
Ll.LtrB 5′ end (-) strand	RT of intron splice junction	CGATTGTCTTTAGGTAACTCAT
Ll.LtrB 3′ end (+) strand	PCR of intron splice junction	CGAAGAGGGTGGTGCAAACCAGT
Ll.LtrB 5′ end (-) strand	PCR of intron splice junction	TTCGGTTAGGTTGGCTGTTTTC
exon2 (-) strand	Poisoned primer extension assay	GCCAGTATAAAGATTCGTAGAAT
Ll.LtrB 3′ end (+) strand	mutagenesis -138	CATAAGCATAAGTGAATTTTTACGAACGAAC
Ll.LtrB 3′ end (-) strand	mutagenesis -138	TATAACATGTCGATGACAATGAAAGCATAC
Ll.LtrB IBS1/IBS2 region (+) strand	mutagenesis GC rich	CGCGGCGTGCGCCCAGATAGGGT
Ll.LtrB IBS1/IBS2 region (-) strand	mutagenesis GC rich	GCCGCGTTCACGATCGACGTGGGTTG
Ll.LtrB EBS1 region (+) strand	mutagenesis GC rich	TAAGTTATGGCCGCGGACTTATCTGTTATCAC
Ll.LtrB EBS1 region (-) strand	mutagenesis GC rich	CCTTTCTTTGTACTAGAGG
Ll.LtrB EBS2 region (+) strand	mutagenesis GC rich	AATTTCGGTTCCGCGTCGATAGAGGAAAG
Ll.LtrB EBS2 region (-) strand	mutagenesis GC rich	AGAAACTTGCGTTCAGTAAAC
Ll.LtrB IBS1/IBS2 region (+) strand	mutagenesis AT rich	TCTATAACGTGCGCCCAGATAG
Ll.LtrB IBS1/IBS2 region (-) strand	mutagenesis AT rich	TATATTCACGATCGACGTGGGT
Ll.LtrB EBS1 region (+) strand	mutagenesis AT rich	AGTTATGGTTATAGACTTATCTGTTATCAC
Ll.LtrB EBS1 region (-) strand	mutagenesis AT rich	TACCTTTCTTTGTACTAGAGG
Ll.LtrB EBS2 region (+) strand	mutagenesis AT rich	TTTCGGTTATATATCGATAGAGGAAAG
Ll.LtrB EBS2 region (-) strand	mutagenesis AT rich	TTAGAAACTTGCGTTCAGTAAAC
Ll.LtrB IBS1/IBS2 region (+) strand	mutagenesis of region IBS2	CGATCGTGAAGTGTGCCATAACGTGCGCCCAGATAG
end of exon1 (-) strand	mutagenesis of region IBS2	ACGTGGGTTGCAATCACA
Ll.LtrB EBS2 region (+) strand	mutagenesis of region EBS2	AATTTCGGTTCACACTCGATAGAGGAAAGTGTCTGAAACCTCTAG
Ll.LtrB EBS2 region (-) strand	mutagenesis of region EBS2	AGAAACTTGCGTTCAGTAAAC
Ll.LtrB EBS1 region (+) strand	mutagenesis of region EBS1	GTAAGTTATGCAACACGACTTATCTGTTATCACCAC
Ll.LtrB EBS1 region (-) strand	mutagenesis of region EBS1	CTTTCTTTGTACTAGAGGTTTC
Ll.LtrB IBS1/IBS2 region (+) strand	mutagenesis of region IBS1	TGAACACATCGTGTTGGTGCGCCCAGATAGGGTGTTAAG
end of exon1 (-) strand	mutagenesis of region IBS1	CGATCGACGTGGGTTGCA
*rlxA* gene (+) strand	cloning in pDL-P23^2^ and pLE-PNis	AAAGCGGCCGCTTATAGTTTGGGCTTAATGTCGG
*rlxA* gene (-) strand	cloning in pDL-P23^2^ and pLE-PNis	AAAGCGGCCGCGTTTAAATGCGCTGGTTCAGAG
*pcfG* gene (+) strand	cloning in pDL-P23^2^ and pLE-PNis	AAAGCGGCCGCATGGTTGTTGTTCCAAAACCTAAG
*pcfG* gene (-) strand	cloning in pDL-P23^2^ and pLE-PNis	AAAGCGGCCGCTTATCTTCTGGCATGTGGTTGAT

### Extraction of total RNA, poisoned primer extension, 3′ RACE and RT-PCR of released intron splice junctions assays

Total RNA was isolated using the standard Trizol-based method as previously described [19, 20] from *L*. *lactis* and *E*. *faecalis* containing various intron constructs expressed either from the bacterial chromosome, the P_23_ constitutive promoter or the nisin-inducible promoter (primers are shown in Table 1). Total RNA extracts were used for poisoned primer extension [4, 21], 3′ RACE [4] and RT-PCR of intron splice junctions assays [5, 22].

**Fig 2:**
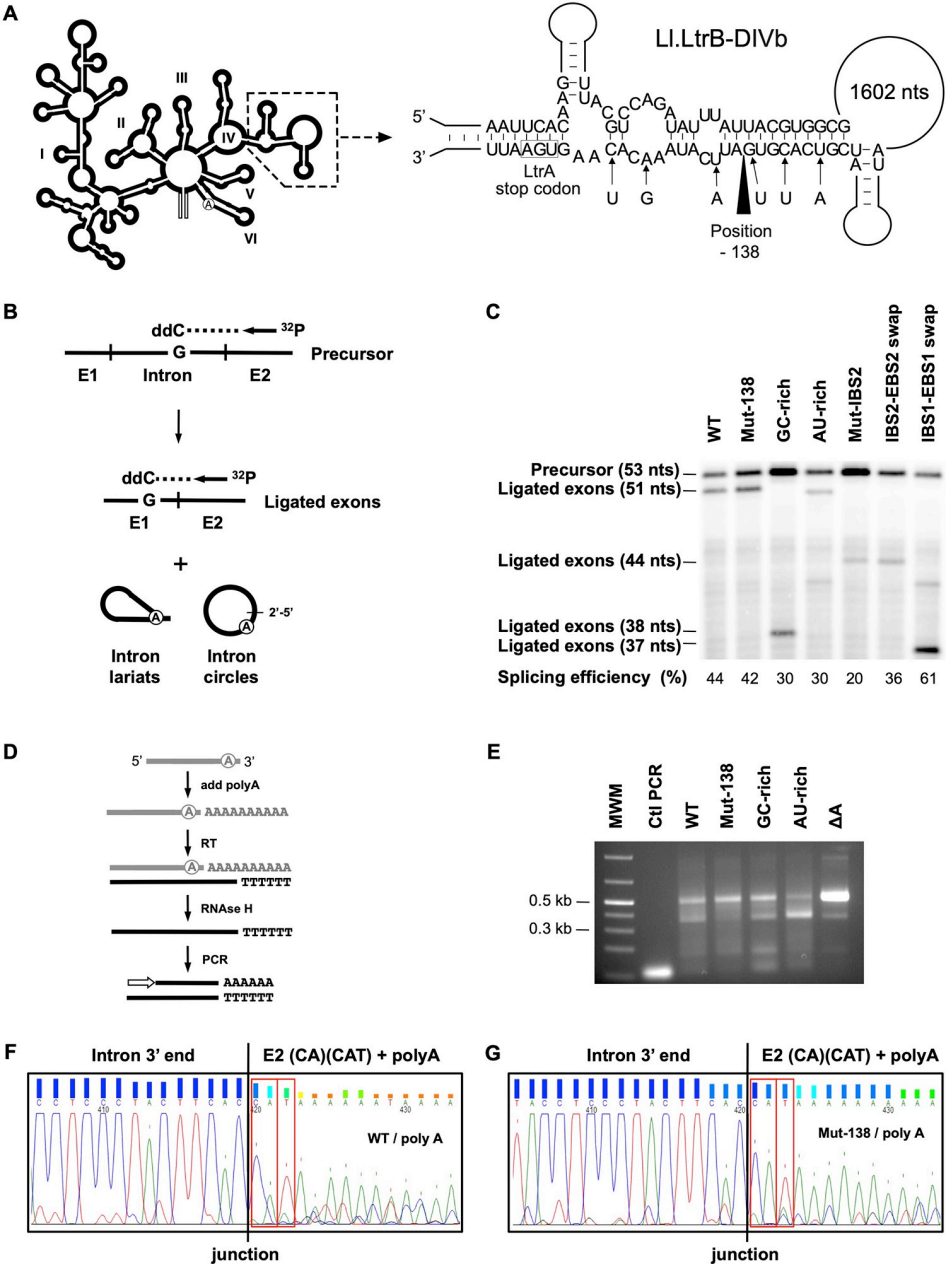
Splicing efficiency and detection of circularization intermediates for various Ll.LtrB constructs. (A) Position of the 6 mutations (arrows) engineered around the -138 processing site (black arrowhead) in DIVb of Ll.LtrB-WT are shown (Ll.LtrB-Mut-138). The position of DIVb relative to the overall Ll.LtrB secondary structure (DI-DVI) is also depicted (dashed area). (B) The splicing efficiency of Ll.LtrB variants expressed from the pDL-P_23_^2^ plasmid was assessed by the poisoned primer extension assay [4, 21]. This assay monitors intron splicing efficiency by comparing the relative abundance of precursor mRNAs (53 nts) and ligated exons (WT, Mut-138 and AU-rich: 51 nts; Mut-IBS2 and IBS2-EBS2 swap: 44 nts; GC-rich: 38 nts; IBS1-EBS1 swap: 37 nts) from total RNA extracts. (C) The poisoned primer extension reactions were run on a 8% PAGE 8M urea gel and splicing efficiency (Splicing efficiency %) was calculated as the relative intensity of the ligated exons (LE) and precursor (P) bands (LE / (P + LE)). (D) Free intron 3′ ends were identified from *L*. *lactis* total RNA extracts by rapid amplification of cDNA 3′ ends (3′ RACE) [4]. The PCR reactions were run on a 2% agarose gel (E). Amplicons corresponding to the circularization intermediates (~500 nts) and the -138 processing site (~350 nts) were all recovered and sequenced. Chromatograms of the circularization intermediates (~500 nts) for WT (F) and Mut-138 (G) are shown.

Splicing efficiency was determined by poisoned primer extension (Fig 2B) [4, 21]. This assay monitors intron splicing by comparing the relative abundance of precursor mRNAs (Precursor) and ligated exons (Ligated exons) from total RNA extracts. A ^32^P-labeled primer (Fig 2B, arrow) (Table 1) complementary to E2 was extended from both the precursor mRNAs and the ligated exons in the presence of a high concentration of ddCTP. Since the sequences of the two RNAs are different after the E2 junction, the first G residue encountered is at a different distance from the primer generating bands of different sizes for the precursor mRNAs (53 nts) and the ligated exons (WT, Mut-138 and AU-rich: 51 nts; Mut-IBS2 and IBS2-EBS2 swap: 44 nts; GC-rich: 38 nts; IBS1-EBS1 swap: 37 nts). The reactions were run on a 8% denaturing 8M urea PAGE gel (Fig 2C) and splicing efficiency (Splicing efficiency %) was calculated as the relative intensity of the ligated exons (LE) and precursor mRNA (P) bands (LE / (P + LE)). The primer was labelled at its 5`end with ^32^P by the T4 polynucleotide enzyme [21]. The intensity of the bands were evaluated using the Quantity One Software from Bio-Rad.

Free intron 3′ ends of the circularization intermediates were identified from *L*. *lactis* total RNA extracts by rapid amplification of cDNA 3′ ends (3′ RACE) (Fig 2D) [4]. Intron 3′ ends were first extended with a polyA tail followed by the synthesis of a cDNA with an oligo dT. The RNA strand was removed by an RNase H treatment and the single strand DNA amplified by PCR. The RT-PCR reactions were run on a 2% agarose gel (Fig 2E). The bands were cut out and recovered from the agarose gel to be directly sequenced (Fig 2F and 2G).

**Fig 3:**
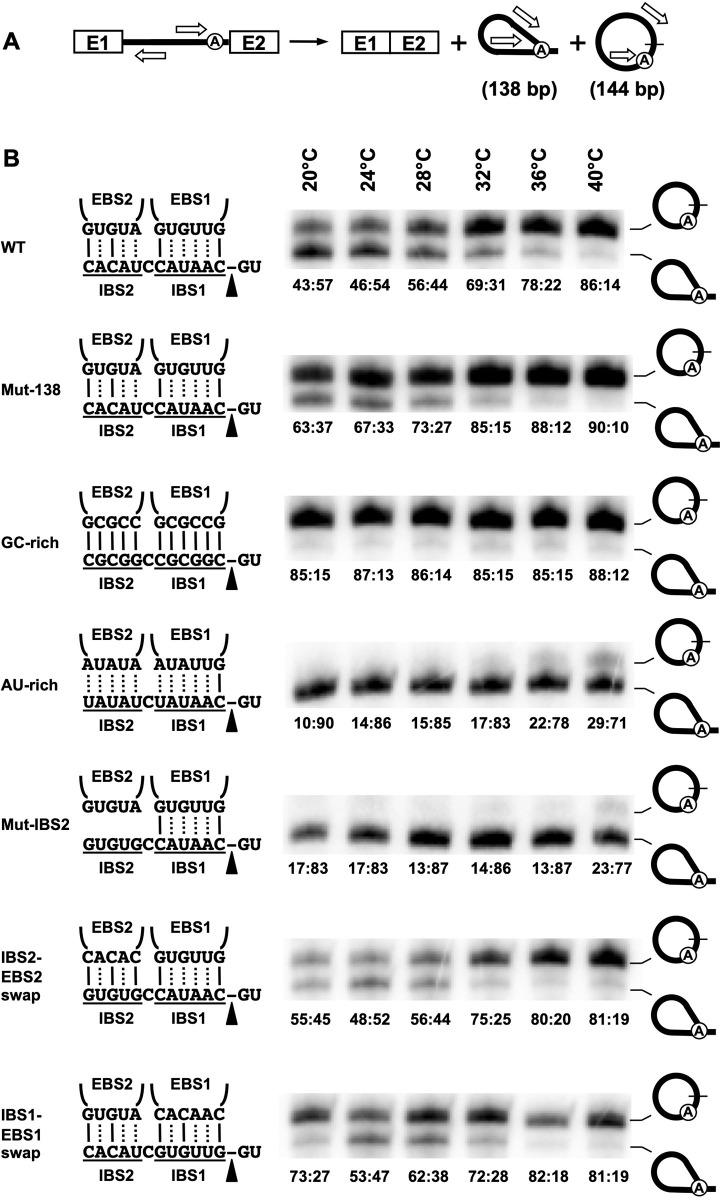
Circle to lariat ratios of various Ll.LtrB constructs at different growth temperatures. (A) Scheme of the combined Ll.LtrB circularization and branching splicing pathways in *L*. *lactis*. Position of the primers used to amplify the excised intron circle (144 bp) and lariat (138 bp) splice junctions by RT-PCR is depicted (open arrows). (B) RT-PCR perfomed on total RNA extracts from *L*. *lactis* grown at various temperatures (20°C to 40°C) and expressing different Ll.LtrB variants from the pDL-P_23_^2^ plasmid. The amplicons were labelled at the 5′ end with ^32^P and ran on 8% denaturing PAGE gels [5]. The IBS1/EBS1 and IBS2/EBS2 base pairing interactions between the intron (EBS1/2) and the 3′ end of E1 (IBS1/2) are shown along with the position of the 5′ splice site (black arrowhead). Two or three hydrogen bonds between complementary nts are denoted by a dotted or solid line respectively. The circle to lariat (circle:lariat) ratios (circle / (circle + lariat):lariat / (lariat + circle) are displayed under each lane.

Intron splice junctions were amplified by RT-PCR (Fig 3A) [5, 22]. The RT-PCR amplicons were labelled at the 5′ end with ^32^P and ran on 8% denaturing 8M urea PAGE gels (Fig 3B) [5]. The intensity of the bands corresponding to excised intron circle (144 bp) and lariat (138 bp) splice junctions were evaluated using the Quantity One Software from Bio-Rad. A boxed area with identical surface was used to evaluate all band intensities even when no apparent bands were present on the image. The circle to lariat (circle:lariat) ratios were calculated as follows: (circle / (circle + lariat):lariat / (lariat + circle).

## Results

### A processing site influences the circle to lariat ratio of Ll.LtrB in *L*. *lactis*

We previously identified by Northern blot and 3′ RACE [4] a processing site in domain IVb (DIVb) of Ll.LtrB-WT at position -138 from the 3′ end of the intron (Fig 2A). While no particular function was previously found associated with this prominent processing site, its presence presumably prevented the detection and analysis of important Ll.LtrB-WT circularization intermediates (Fig 1B, step 1) by 3′ RACE [4]. In contrast, when most of the *ltrA* gene is missing from DIV, the intron was found to be no longer processed at position -138 regardless of whether the LtrA protein was co-expressed *in trans* (Ll.LtrB-ΔLtrA+LtrA) or not (Ll.LtrB-ΔLtrA) [4]. We thus hypothesized that a structural motif in DIVb is specifically recognized and cleaved by an *L*. *lactis* RNase [4] and that removal of nearly all of the *ltrA* gene in the vicinity of that motif modified its structure in a way that precluded its recognition and cleavage.

To prevent the formation of that putative RNase recognition motif within Ll.LtrB-WT, we introduced 6 point mutations, 3 on each side of the processing site (Fig 2A, Ll.LtrB-Mut-138). We mutated the third nt of 6 *ltrA* codons to disrupt the structural motif while maintaining the amino acid sequence of the expressed LtrA protein. Ll.LtrB-Mut-138 was first shown to splice accurately by RT-PCR of both ligated exons and released introns [4, 5] and as efficiently as Ll.LtrB-WT by poisoned primer extension (42% *vs* 44%) (Fig 2B and 2C) [4, 21]. To compare the circularization intermediates of Ll.LtrB-WT and Ll.LtrB-Mut-138 we performed 3′ RACE assays on *L*. *lactis* total RNA extracts (Fig 2D) [4]. The intron 3′ ends were first extended with a polyA tail followed by the synthesis of a cDNA with an oligo dT. The RNA strand from the RNA/DNA duplex was then removed and the cDNA was amplified by PCR. As previously observed, a strong circularization intermediate band (~500 nt) was obtained for Ll.LtrB-ΔA (Fig 2E) that splices exclusively through the circularization pathway (Fig 1B) [4, 5]. The band corresponding to the -138 processing site (~350 nt) was absent for Ll.LtrB-Mut-138 while the circularization intermediate band was slightly more intense compared to Ll.LtrB-WT (Fig 2E). The circularization intermediate amplicons for both introns were sequenced and shown to harbor at their 3′ end the previously identified CA and CAU tails (Fig 2F and 2G). These additional nts at the 3′ end of the intron were formely shown to correspond to the first residues of E2 generated by misrecognition of the 3′ splice site during the first step of circularization (Fig 1B, step 1) [4].

RT enzymes do not progress through the 2′-5′ bond at the branch point of lariats as efficiently as through the intron circle junction, causing a bias that likely underestimates the amount of intron lariats [4, 5, 23]. Since the absolute abundance of intron circles and lariats is difficult to determine precisely, we next decided to compare, for both introns, the relative circle to lariat ratio (circle:lariat) (circle / (circle + lariat):lariat / (lariat + circle). Amplification of the released intron splice junctions by RT-PCR [5] showed that the relative amount of Ll.LtrB-Mut-138 circles compared to lariats (57.5:42.5) is slightly higher than for Ll.LtrB-WT (47.2:52.8) even though they both display the same splicing efficiency (Fig 2C) [4, 21].

These results show that the presence of an important processing site in DIVb does not affect the overall splicing efficiency and accuracy of Ll.LtrB-WT, yet influences the circle to lariat ratio *in vivo*. In addition, the detection of circularization intermediates with CA and CAU tails for both Ll.LtrB-Mut-138 and Ll.LtrB-WT further validates the group II intron circularization pathway previously characterised [4] (Fig 1B) and confirms that branching and circularization both coexist as significant and conserved splicing pathways in *L*. *lactis* [4, 5].

### The circle to lariat ratio of Ll.LtrB is affected by growth temperature and the strength of the base pairing interactions between the intron and its upstream exon

We performed RT-PCR assays accross the intron splice junctions from total RNA extracts of *L*. *lactis* to evaluate the influence of various biological factors on the circle to lariat ratio of Ll.LtrB *in vivo* (Fig 3). Group II intron splicing was previously shown to be influenced by temperarure both *in vitro* and *in vivo* [24–26]. We thus first investigated various growth temperatures below and above 30ºC, the optimal growth temperature of *L*. *lactis*. The cultures were grown at 4ºC intervals ranging from 20ºC to 40ºC. Next, we assessed the effect of the strength of the base pairing interactions between the intron and its upstream exon since recognition of the 5`splice site is different for the branching and circularization pathway (Fig 1). All Ll.LtrB variants studied were first shown to splice accurately by RT-PCR of both ligated exons and released introns [4, 5] and efficiently by poisoned primer extension (Fig 2B and 2C) [4, 21].

The relative proportion of circles over lariats was found to progressively increase with temperature for Ll.LtrB-WT and Ll.LtrB-Mut-138 where the overall amount of circles were consistently higher for Ll.LtrB-Mut-138 (Fig 3B). Next, the base pairing interactions between EBS1/2 and IBS1/2, that are used by the intron to identify the E1-intron junction at the 5′ splice site, were either strengthened (Ll.LtrB-GC-rich) or weakened (Ll.LtrB-AU-rich) to assess their influence on the Ll.LtrB circle to lariat ratio. We found that the Ll.LtrB-GC-rich is mostly released as circles while, in contrast, the Ll.LtrB-AU-rich is primarly found as lariats (Fig 3B). The proportion of circles slightly increased with temperatures from 10% (10:90) to 29% (29:71) for Ll.LtrB-AU-rich but stayed constantly high at around 85% (85:15) for Ll.LtrB-GC-rich at all temperatures. Accordingly, analysis of the intron extremities by 3′ RACE showed fewer circularization intermediates (~500 nt) for Ll.LtrB-AU-rich, leading to the detection of more processed introns (~350 nt) when compared to Ll.LtrB-GC-rich (Fig 2E).

We then weakened the recognition of the 5′ splice site by the intron using a different approach. The IBS2-EBS2 base pairing interaction was completely abrogated by mutating the IBS2 sequence of E1 (Ll.LtrB-Mut-IBS2). Ll.LtrB-Mut-IBS2 behaved like the Ll.LtrB-AU-rich construct showing reduced splicing efficiency (Fig 2C) and low levels of circles that only slightly increased from 17% (17:83) to 23% (23:77) with temperature (Fig 3B). Restoration of the IBS2-EBS2 base pairing interaction by complementation (Ll.LtrB-IBS2-EBS2 swap) led to full recovery of the wild-type splicing efficiency (Fig 2C), circle to lariat ratios and temperature sensitivity phenotypes (Fig 3B). On the other hand, weakening the recognition of E1 by mutating the EBS1 sequence of the intron (Ll.LtrB-Mut-EBS1) totally inhibited splicing. This was expected since the IBS1/EBS1 interaction is more important than the IBS2/EBS2 interaction being located right next to the E1-intron splice junction [27]. Nevertheless, similarly to the Ll.LtrB-IBS2-EBS2 swap construct, restoration of the base pairing interaction by complementation (Ll.LtrB-IBS1-EBS1 swap) led to full recovery of the splicing efficiency (Fig 2C), circle to lariat ratios and temperature sensitivity phenotypes (Fig 3B).

Collectively, these findings show that growth temperature as well as the strength of the base pairing interaction between the intron (EBS1/2) and the 3′ end of E1 (IBS1/2) at the 5′ splice site have considerable influences on the splicing efficiency and overall circle to lariat ratios of Ll.LtrB during *L*. *lactis* exponential growth.

### The cellular environment and expression level of interrupted relaxase genes influence the circle to lariat ratio of their released group II introns

**Fig 4:**
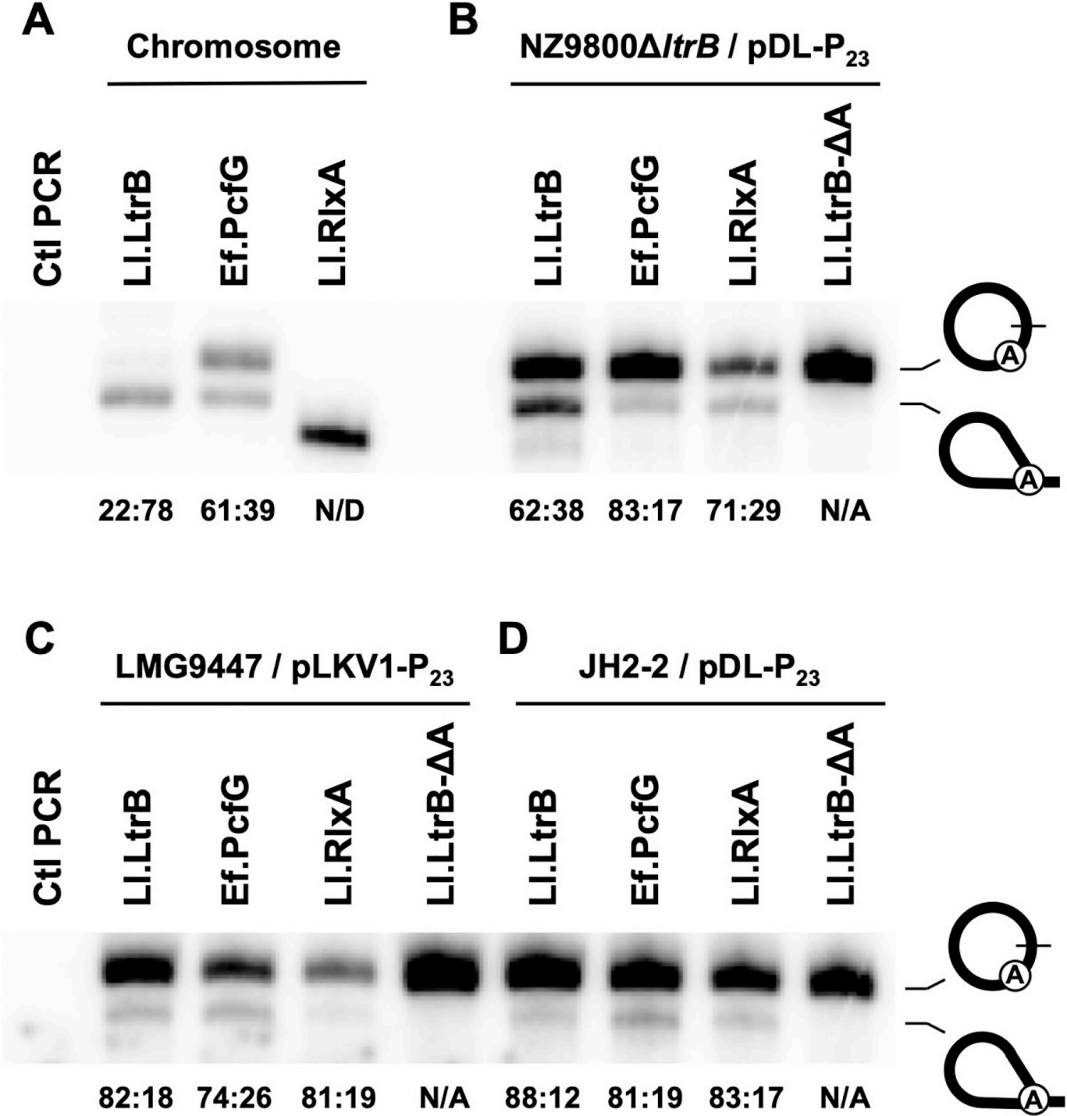
Circle to lariat ratios of three bacterial group II introns in various cellular environments. RT-PCR amplification of excised intron junctions was perfomed on total RNA extracts of bacterial cells expressing intron-interrupted relaxase genes in their native environments (A) (*ltrB* gene from *L*. *lactis* strain NZ9800 (Ll.LtrB intron); *pcfG* gene from *Enterococcus faecalis* strain SF24397 (Ef.PcfG intron); *rlxA* gene from *L*. *lactis* strain LMG9447 (Ll.RlxA intron)), and also from the P_23_ constitutive promoter in *L*. *lactis* NZ9800Δ*ltrB* (B), in *L*. *lactis* LMG9447 (C) and in *E*. *faecalis* JH2-2 (D). The branch point variant of Ll.LtrB (Ll.LtrB-ΔA) was also expressed from the P_23_ constitutive promoter in the three host environments (B-D). The circle to lariat (circle:lariat) ratios (circle / (circle + lariat):lariat / (lariat + circle) are displayed under each lane. The lower band in the Ll.RlxA lane (panel A) was sequenced and corresponds to the non-specific amplification of the unspliced precursor mRNA (intron 3′ end-Exon 2). N/D (not detected): no intron circle and lariat detected. N/A (not applicable): Ll.LtrB-ΔA splices exclusively through circularization.

The circle to lariat ratios were compared for three bacterial group II introns interrupting different orthologous relaxase genes: Ll.LtrB and Ll.RlxA from two strains of *L*. *lactis* (NZ9800, LMG9447) and Ef.PcfG from *Enterococcus faecalis* (SF24397) (Fig 4). While Ll.LtrB and Ef.PcfG were shown to use both splicing pathways when expressed from their natural promoter in their native environment, no trace of lariats or circles was detected for Ll.RlxA (Fig 4A). In fact, the lower RT-PCR band detected for Ll.RlxA was sequenced and corresponds to the 3′ splice junction of the unspliced pre-mRNA showing that the *rlxA*-interrupted gene is indeed expressed but that the intron does not splice by either pathway in native conditions. The level of released circles was found to be higher for Ef.PcfG in *E*. *faecalis* at (61%) (61:39) compared to Ll.LtrB in *L*. *lactis* (22%) (22:78) mainly due to the presence of more Ef.PcfG circles.

Expression of the three intron-interrupted relaxase genes from the pDL plasmid in *L*. *lactis* (NZ9800Δ*ltrB*) under the control of the P_23_ constitutive promoter increased the overall level of released introns compared to their native promoters and considerably changed their circle to lariat ratios (Fig 4B). The proportion of released intron circles of Ll.LtrB and Ef.PcfG increased by 40% and 22% respectively, primarly because of the presence of more intron circles. Interestingly, Ll.RlxA, which was found to be inactive in its *L*. *lactis* native environment (LMG9447), was shown to use both splicing pathways when overexpressed in a different *L*. *lactis* strain exhibiting a circle to lariat ratio intermediate (71:29) to Ll.LtrB (62:38) and Ef.PfcG (83:17).

The circle to lariat ratio of the three introns was also assessed in LMG9447, the *L*. *lactis* strain naturally carrying the Ll.RlxA intron (Fig 4C). Fewer lariats of Ll.LtrB were detected when compared to overexpression in its native environment (Fig 4B), leading to a 20% increase in the proportion of released intron circles. In contrast, the level of Ef.PcfG circle is 9% lower in LMG9447 compared to NZ9800Δ*ltrB* mainly due to the presence of fewer circles. In the case of Ll.RlxA, that was found to be inactive in native conditions (Fig 4A), overexpression of the *rlxA*-interrupted gene from a constitutive promoter in its original strain stimulates both splicing pathways. The relative amount of Ll.RlxA circles is 10% higher in LMG9447 compared to NZ9800Δ*ltrB* mainly because of a reduction in lariats.

Once again, compared to their native environments (Fig 4A), high circle to lariat ratios were observed when the three intron-interrupted genes were overexpressed from the P_23_ promoter in a lab strain of *Enterococcus faecalis* (JH2-2) (Fig 4D). Contrarily to the three wild-type introns, Ll.LtrB-ΔA, that was previously shown to splice exclusively through the circularization pathway in NZ9800Δ*ltrB* [4, 5], only generates intron circles regardless of the host environment (Fig 4B–4D).

**Fig 5:**
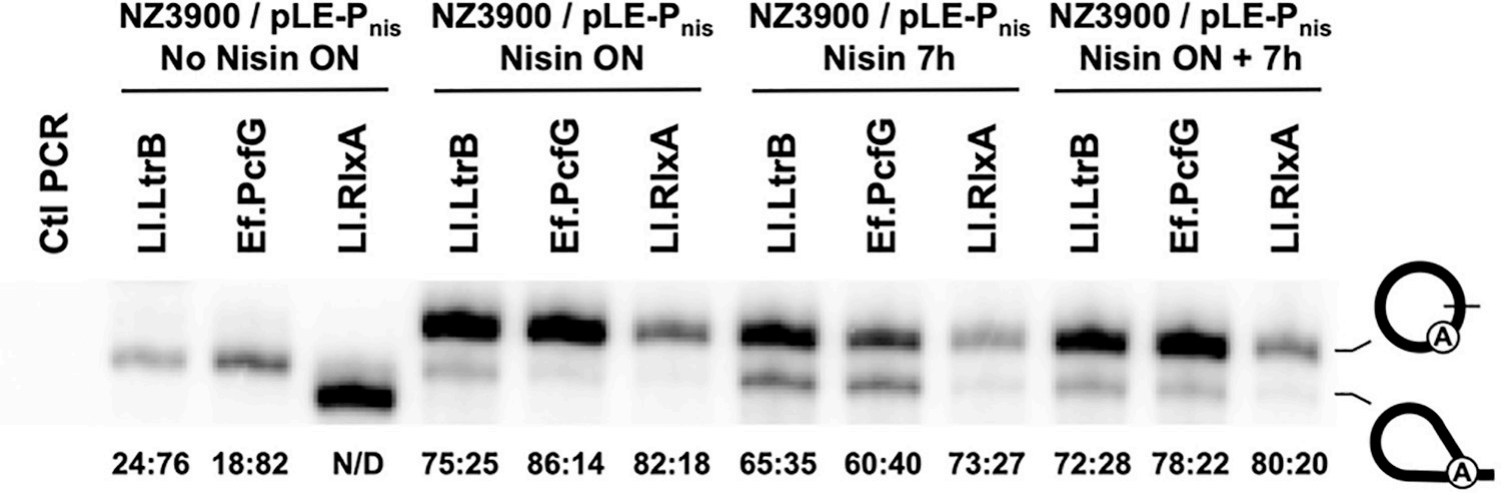
Circle to lariat ratios of three bacterial group II introns expressed at different levels in *L*. *lactis*. RT-PCR amplification of excised intron junctions was perfomed on total RNA extracts of the NZ3900 *L*. *lactis* strain expressing three intron-interrupted relaxase genes (*ltrB* gene from *L*. *lactis* strain NZ9800 (Ll.LtrB intron); *pcfG* gene from *Enterococcus faecalis* strain SF24397 (Ef.PcfG intron); *rlxA* gene from *L*. *lactis* strain LMG9447 (Ll.RlxA intron)) from the strong nisin-inducible promoter (P_nis_). The circle to lariat (circle:lariat) ratios (circle / (circle + lariat):lariat / (lariat + circle) are displayed under each lane. N/D (not detected): no intron circle and lariat detected. The lower band in the Ll.RlxA lane was sequenced and corresponds to the non-specific amplification of the unspliced precursor mRNA (intron 3′ end-Exon 2). Induction conditions: non-induced overnight culture, No Nisin ON; nisin-induced overnight culture, Nisin ON; 7h exponential growth nisin-induced culture, Nisin 7h; nisin-induced overnight culture followed by 7h exponential growth nisin-induced culture, Nisin ON + 7h.

Next, the nisin-inducible promoter (P_nis_) was used to express the three intron-interrupted genes from the pLE plasmid in a different strain of *L*. *lactis* (NZ3900) for either overnight, 7h, or overnight followed by a second induction period of 7h (Fig 5). For the uninduced controls, the expression levels and splicing phenotypes of Ll.LtrB and Ll.RlxA are comparable to when they are expressed from their natural promoter in their native environment (Fig 4A). In contrast, very few Ef.PcfG circles are detected, leading to a substantial shift of the circle to lariat ratio from 61:39 to 18:82. Overall, similar levels of released introns were detected for all three nisin-induced expression conditions (Fig 5) and comparable to the amount of intron expressed from the P_23_ constitutive promoter (Fig 4B–4D). In general, only small differences of the circle to lariat ratios were detected between the P_23_ (Fig 4B–4D) and P_nis_ (Fig 5) overexpression conditions. The variations in circle to lariat ratio are again largely due to the accumulation of intron circles since lariat levels are comparable for the same intron between the three nisin induction conditions. This is in accordance with previously published data suggesting that group II intron circles slowly accumulate with time following the induction of their intron-interrupted genes [5].

Collectively, these results demonstrate that the expression level of intron-interrupted genes and the cellular environment both have a great influence on the circle to lariat ratios of bacterial group II introns *in vivo*. Moreover, we found that similarly to Ll.LtrB and Ll.RlxA in *L*. *lactis*, the Ef.PcfG group II intron uses the branching and circularization pathways concurrently in *E*. *faecalis* as well as in various strains of *L*. *lactis*. We also show that the two *L*. *lactis* group II introns use both splicing pathways in *E*. *faecalis*.

## Discussion

Taking advantage of the Ll.LtrB branch point mutant, that was previously shown to splice exclusively as circles [5], we characterized the circularization pathway of bacterial group II introns *in vivo* [4, 5]. In addition, we demonstrated that all variants of Ll.LtrB-WT studied spliced using the circularization and branching pathways *in vivo*, leading to the accumulation of significant amounts of both intron circles and lariats [4, 5].

Even though we clearly identified, for various Ll.LtrB constructs, the same splicing intermediates that support the proposed circularization pathway, we were unable to detect them for Ll.LtrB-WT [4]. The presence of a prominent processing site, located towards the end of the *ltrA* gene at position -138 from the 3′ end of the intron, seemed to prevent the detection of circularization intermediates [4]. We thus engineered Ll.LtrB-Mut-138 (Fig 2A), a variant of Ll.LtrB-WT that was first shown to be no longer processed at position -138 (Fig 2E) and that allowed detection of the circularization intermediates (Fig 2E and 2G). Moreover, following minor adjustments of the 3`RACE protocol, the same circularization intermediates were also ultimately detected for Ll.LtrB-WT (Fig 2E and 2F). These data strongly corroborate the group II intron circularization pathway previously characterised [4] and confirm that branching and circularization both co-exsist as significant, conserved and competing splicing pathways in *L*. *lactis* [4, 5].

Since Ll.LtrB can self-splice through two significant competing pathways in *L*. *lactis* we decided to further address the factors that might influence the circle to lariat ratio of excised group II introns in bacteria. We first noticed that removal of the processing site in DIVb increased the proportion of released Ll.LtrB-Mut-138 circles compared to Ll.LtrB-WT by approximately 10% when *L*. *lactis* was grown at 30°C. Moreover, a small but steady accumulation of Ll.LtrB-WT circles was previously observed during *L*. *lactis* exponential growth at 30°C while lariat levels remained relatively constant throughout the growth curve [5]. These results suggest that stability of the released introns and growth phase of its host both affect the circle to lariat ratio of Ll.LtrB *in vivo*. Although we cannot rule out completely a reduction in lariats to explain the relative increase of intron circles, removal of a processing site within the intron is more likely to stabilize the splicing intermediates and/or the released intron circles than to reduce the accumulation of lariats. This is concurring with the fact that intron circles are dead end spliced products that can only accumulate since they cannot reverse splice through the circularization pathway (Fig 1B). In contrast, released intron lariats are active ribozymes that can invade various RNA [6] and/or DNA [17, 28] substrates by reverse splicing through the branching pathway (Fig 1A).

We then investigated the effect of temperature on the circle to lariat ratio of Ll.LtrB. In general, we found that *L*. *lactis* growth temperature between 20°C and 40°C influences the circle to lariat ratio of all the Ll.LtrB variants studied, albeit at various levels, without significantly affecting their overall splicing efficiencies. Our data show an overall steady increase of the proportion of intron circles with temperature (Fig 3). Differences in circle to lariat ratios between Ll.LtrB-WT and Ll.LtrB-Mut-138 were noticed at all temperatures however decreasing from 21% to 4% as the growth temperature increased (Fig 3). The differences in circle to lariat ratios between Ll.LtrB-WT and Ll.LtrB-Mut-138 seem to come from the accumulation of more Ll.LtrB-Mut-138 circles. Our data thus suggest that higher temperatures favor the circularization pathway for all Ll.LtrB variants studied. The first transesterification reaction of the group II intron circularization pathway is initiated *in trans* by the 3′-OH of free E1 (Fig 1B, step 1). Free E1 is most likely in competition with *cis* E1 to base pair with the EBS1/2 sequences of the unspliced intron in the pre-mRNA. We thus assume that higher temperatures facilitate the access of free E1 to base pair with the EBS1/2 of the intron and/or favoring the displacement of *cis* E1 without significantly affecting splicing efficiency. Higher temperatures may also lead to more efficient release of free E1 from the intron after the first step of the branching pathway (Fig 1A, step 1) having the effect of both reducing branching rates and inducing circularization.

Next, we assessed whether the strength of the base pairing interactions between the intron (EBS1/2) and the 3′ end of the *cis* E1 (IBS1/2) and free E1 (IBS1/2) influence the circle to lariat ratio of Ll.LtrB *in vivo*. Strengthening of the base pairing interaction between the intron and E1 led to an important increase of the proportion of released intron circles compared to lariats, independently of temperature (Fig 3, Ll.LtrB-GC-rich). In contrast, weakening the interaction between the intron and E1 by either decreasing the overall strength of the base pairing interactions (Fig 3, Ll.LtrB-AU-rich) or by completely abrogating the EBS2-IBS2 interaction (Fig 3, Ll.LtrB-Mut-IBS2) led to substantial decreases of the proportion of released intron circles compared to lariats that slightly increased with temperature. Taken together, these data suggest that strengthening of the EBS-IBS interactions enhances intron circularization while weakening of these interactions between E1 and the intron reduces circularization efficiency. Nevertheless, we cannot rule out completely a reduction in branching efficiency to explain increases in relative amounts of intron circles following the strengthening of the EBS-IBS interactions.

The level of expression of intron-interrupted genes was also shown to have a significant effect on the circle to lariat ratio of three different group II introns from two bacterial species. Increasing the expression compared to natural expression levels in their original host environments led to important increases in the relative amounts of released of intron circles for Ll.LtrB and Ll.RlxA from *L*. *lactis*, and Ef.PcfG from *E*. *faecalis*. The increase in released circles were seen in both *L*. *lactis* and *E*. *faecalis* regardless if a constitutive (P_23_) (Fig 4) or an inducible (Nisin) (Fig 5) promoter was used and appeared to come from the accumulation of circles and/or the stimulation of the circularization pathway. Our data also demonstrated that branching and circularization are used concurrently in both *L*. *lactis* and *E*. *faecalis* by the three different introns.

Interestingly, no trace of circles or lariats were observed for the Ll.RlxA intron in its *L*. *lactis* host strain even though the interrupted pre-mRNA can be readily detected (Fig 4A). This demonstrated that the interrupted gene is indeed expressed in LMG9447 but that the intron cannot splice by either circularization or branching in native expression conditions. However, Ll.RlxA circles and lariats were detected when the interrupted-*rlxA* gene was overexpressed in the NZ9800Δ*ltrB* (Fig 4B) and NZ3900 (Fig 5) *L*. *lactis* strains and the JH2-2 *E*. *faecalis* strain (Fig 4D) indicating that the intron is splicing competent. Ll.RlxA was also shown to be splicing competent in its native environment when overexpressed (Fig 4C). Similarly to Ll.LtrB and Ef.PcfG, the proportion of released Ll.RlxA circles compared to lariats is high and slightly different in the four bacterial strains studied varying from 71:29 to 83:17. Taken together these results show that Ll.RlxA cannot splice by either pathway when the interrupted gene is expresessed at lower levels from its natural promoter. In accordance, no Ll.RlxA splicing was detected under the no nisin condition in the NZ3900 *L*. *lactis* strain (Fig 5, No Nisin ON). The speed of transcription was previously shown to affect the folding pathways and function of various ribozymes [29, 30]. Overexpression of the interrupted gene in LMG9447 and NZ3900 may thus induce proper folding of the Ll.RlxA intron compared to low expression level and allow splicing through both branching and circularization pathways. Despite the fact that Ll.LtrB and Ef.PcfG are almost identical (99.7%) they behave quite differently in their respective host environment where they can be found mostly as lariats and circles respectively. Overall, our data indicate that cell host environment has an impact on the circle to lariat ratio for the three introns studied. However, even though the three introns are highly similar at the sequence level, being between 95%-99.7% identical, we cannot completely rule out a sequence specific contribution.

Following the transcription of intron-interrupted genes in bacterial cells, group II introns can splice from their pre-mRNA transcripts by using either the branching or the circularization pathway. In this study we demonstrated that various factors like intron stability, growth phase, growth temperature of host cells, expression levels of the intron-interrupted gene, host cell environment, and the strength of the EBS-IBS base pairing interactions between the intron and E1 all have a significant impact on the overall circle to lariat ratio of group II introns *in vivo*. These variations in circle to lariat ratios can be attributed to either a change in splicing efficiencies between branching and circularization and/or differences in accumulation between intron lariats and circles. However, in light of the various factors involved, we presume that the overall circle to lariat ratios observed in bacterial cells are a combination of all these factors and that the observed variations of circle to lariat ratios may affect group II intron function *in vivo*. For example, an important shift from branching to circularization may have a significant effect on group II intron mobility efficiency since the amount of active RNPs harboring intron lariats would be reduced and replaced by intron circles containing RNPs that cannot reverse splice and are thus considered as non-mobile dead-end spliced products.

## Supporting information

S1 Raw images (PDF)

## References

[1] McNeil BA, Semper C, Zimmerly S. Group II introns: versatile ribozymes and retroelements. Wiley Interdiscip Rev RNA. 2016. 10.1002/wrna.1339 26876278

[2] Lambowitz AM, Zimmerly S. Group II introns: mobile ribozymes that invade DNA. Cold Spring Harb Perspect Biol. 2011;3(8):a003616 10.1101/cshperspect.a003616 20463000 PMC3140690

[3] Fedorova O, Zingler N. Group II introns: structure, folding and splicing mechanism. Biological chemistry. 2007;388(7):665–78. 10.1515/BC.2007.090 17570818

[4] Monat C, Cousineau B. Circularization pathway of a bacterial group II intron. Nucleic Acids Res. 2016;44(4):1845–53. 10.1093/nar/gkv1381 26673697 PMC4770220

[5] Monat C, Quiroga C, Laroche-Johnston F, Cousineau B. The Ll.LtrB intron from Lactococcus lactis excises as circles in vivo: insights into the group II intron circularization pathway. Rna. 2015;21(7):1286–93. 10.1261/rna.046367.114 25956521 PMC4478347

[6] LaRoche-Johnston F, Monat C, Coulombe S, Cousineau B. Bacterial group II introns generate genetic diversity by circularization and trans-splicing from a population of intron-invaded mRNAs. PLoS Genet. 2018;14(11):e1007792 10.1371/journal.pgen.1007792 30462638 PMC6248898

[7] Lambowitz AM, Belfort M. Mobile Bacterial Group II Introns at the Crux of Eukaryotic Evolution. Microbiol Spectr. 2015;3(1). 10.1128/microbiolspec.MDNA3-0050-2014 PMC4394904 26104554

[8] Chalamcharla VR, Curcio MJ, Belfort M. Nuclear expression of a group II intron is consistent with spliceosomal intron ancestry. Genes Dev. 2010;24(8):827–36. 10.1101/gad.1905010 20351053 PMC2854396

[9] Curcio MJ, Belfort M. The beginning of the end: links between ancient retroelements and modern telomerases. Proc Natl Acad Sci U S A. 2007;104(22):9107–8. 10.1073/pnas.0703224104 17517612 PMC1890453

[10] Malik HS, Burke WD, Eickbush TH. The age and evolution of non-LTR retrotransposable elements. Mol Biol Evol. 1999;16(6):793–805. 10.1093/oxfordjournals.molbev.a026164 10368957

[11] Bush SJ, Chen L, Tovar-Corona JM, Urrutia AO. Alternative splicing and the evolution of phenotypic novelty. Philos Trans R Soc Lond B Biol Sci. 2017;372(1713). 10.1098/rstb.2015.0474 PMC5182408 27994117

[12] Cordaux R, Batzer MA. The impact of retrotransposons on human genome evolution. Nat Rev Genet. 2009;10(10):691–703. 10.1038/nrg2640 19763152 PMC2884099

[13] Dai L, Zimmerly S. Compilation and analysis of group II intron insertions in bacterial genomes: evidence for retroelement behavior. Nucleic Acids Res. 2002;30(5):1091–102. 10.1093/nar/30.5.1091 11861899 PMC101233

[14] LaRoche-Johnston F, Monat C, Cousineau B. Recent horizontal transfer, functional adaptation and dissemination of a bacterial group II intron. BMC Evol Biol. 2016;16(1):223 10.1186/s12862-016-0789-7 27765015 PMC5072309

[15] Wagner A. Periodic extinctions of transposable elements in bacterial lineages: evidence from intragenomic variation in multiple genomes. Mol Biol Evol. 2006;23(4):723–33. 10.1093/molbev/msj085 16373392

[16] Pyle AM. The tertiary structure of group II introns: implications for biological function and evolution. Crit Rev Biochem Mol Biol. 2010;45(3):215–32. 10.3109/10409231003796523 20446804 PMC4408542

[17] Ichiyanagi K, Beauregard A, Lawrence S, Smith D, Cousineau B, Belfort M. Retrotransposition of the Ll.LtrB group II intron proceeds predominantly via reverse splicing into DNA targets. Mol Microbiol. 2002;46(5):1259–72. 10.1046/j.1365-2958.2002.03226.x 12453213

[18] McBride SM, Fischetti VA, Leblanc DJ, Moellering RC Jr., Gilmore MS Genetic diversity among Enterococcus faecalis. PloS one. 2007;2(7):e582 10.1371/journal.pone.0000582 17611618 PMC1899230

[19] Belhocine K, Mandilaras V, Yeung B, Cousineau B. Conjugative transfer of the Lactococcus lactis sex factor and pRS01 plasmid to Enterococcus faecalis. FEMS microbiology letters. 2007;269(2):289–94. 10.1111/j.1574-6968.2007.00641.x 17263841

[20] Belhocine K, Mak AB, Cousineau B. Trans-splicing of the Ll.LtrB group II intron in Lactococcus lactis. Nucleic Acids Res. 2007;35(7):2257–68. 10.1093/nar/gkl1146 17389638 PMC1874635

[21] Plante I, Cousineau B. Restriction for gene insertion within the Lactococcus lactis Ll.LtrB group II intron. Rna. 2006;12(11):1980–92. 10.1261/rna.193306 16973892 PMC1624911

[22] Belhocine K, Mak AB, Cousineau B. Trans-splicing versatility of the Ll.LtrB group II intron. Rna. 2008;14(9):1782–90. 10.1261/rna.1083508 18648072 PMC2525943

[23] Lorsch JR, Bartel DP, Szostak JW. Reverse transcriptase reads through a 2'-5'linkage and a 2'-thiophosphate in a template. Nucleic Acids Res. 1995;23(15):2811–4. 10.1093/nar/23.15.2811 7544885 PMC307115

[24] Adamidi C, Fedorova O, Pyle AM. A group II intron inserted into a bacterial heat-shock operon shows autocatalytic activity and unusual thermostability. Biochemistry. 2003;42(12):3409–18. 10.1021/bi027330b 12653544

[25] Toor N, Robart AR, Christianson J, Zimmerly S. Self-splicing of a group IIC intron: 5' exon recognition and alternative 5' splicing events implicate the stem-loop motif of a transcriptional terminator. Nucleic Acids Res. 2006;34(22):6461–71. 10.1093/nar/gkl820 17130159 PMC1702495

[26] Dong X, Qu G, Piazza CL, Belfort M. Group II intron as cold sensor for self-preservation and bacterial conjugation. Nucleic Acids Res. 2020;48(11):6198–209. 10.1093/nar/gkaa313 32379323 PMC7293003

[27] Jacquier A, Michel F. Multiple exon-binding sites in class II self-splicing introns. Cell. 1987;50(1):17–29. 10.1016/0092-8674(87)90658-1 3297351

[28] Yang J, Zimmerly S, Perlman PS, Lambowitz AM. Efficient integration of an intron RNA into double-stranded DNA by reverse splicing. Nature. 1996;381(6580):332–5. 10.1038/381332a0 8692273

[29] Pan T, Sosnick T. RNA folding during transcription. Annu Rev Biophys Biomol Struct. 2006;35:161–75. 10.1146/annurev.biophys.35.040405.102053 16689632

[30] Zhao C, Rajashankar KR, Marcia M, Pyle AM. Crystal structure of group II intron domain 1 reveals a template for RNA assembly. Nat Chem Biol. 2015;11(12):967–72. 10.1038/nchembio.1949 26502156 PMC4651773

