# Supplementary material for: The circle to lariat ratio of the Ll.LtrB group II intron from Lactococcus lactis is greatly influenced by a variety of biological determinants in vivo
Source: PLoS One. 2020 Aug 18;15(8):e0237367. doi: 10.1371/journal.pone.0237367 (PMC7444581; doi:10.1371/journal.pone.0237367)

Fig 2C

- WT
- Mut-138
- GC-rich
- AU-rich
- Mut-IBS2
- IBS2-EBS2 swap
- IBS1-EBS1 swap

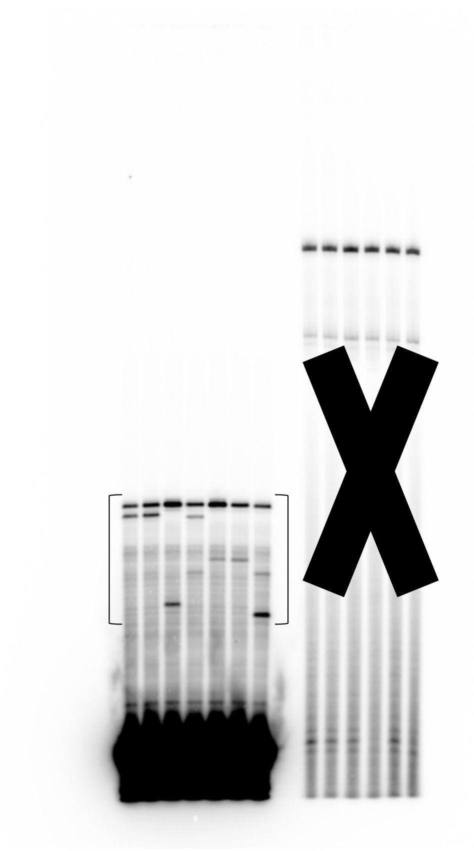

Fig 2E

- MWM
- Ctl PCR
- WT
- Mut-138
- GC-rich
- AU-rich
- ΔA

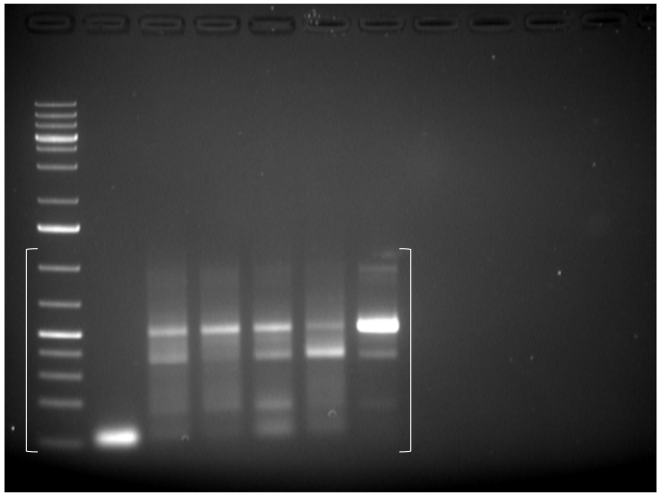

Fig 3B: WT

- 20℃
- 24℃
- 28℃
- 32℃
- 36℃
- 40℃

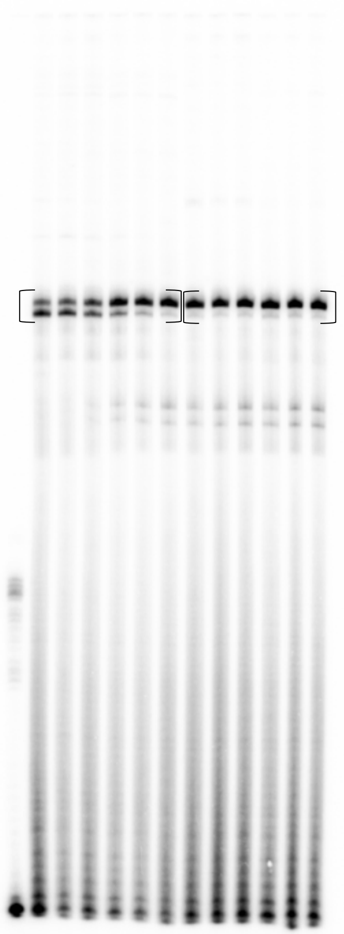

Fig 3B: GC-rich

- 20℃
- 24℃
- 28℃
- 32℃
- 36℃
- 40℃

<u>Fig 3B: Mut-138</u>

- 20℃
- 24℃
- 28℃
- 32℃
- 36℃
- 40℃

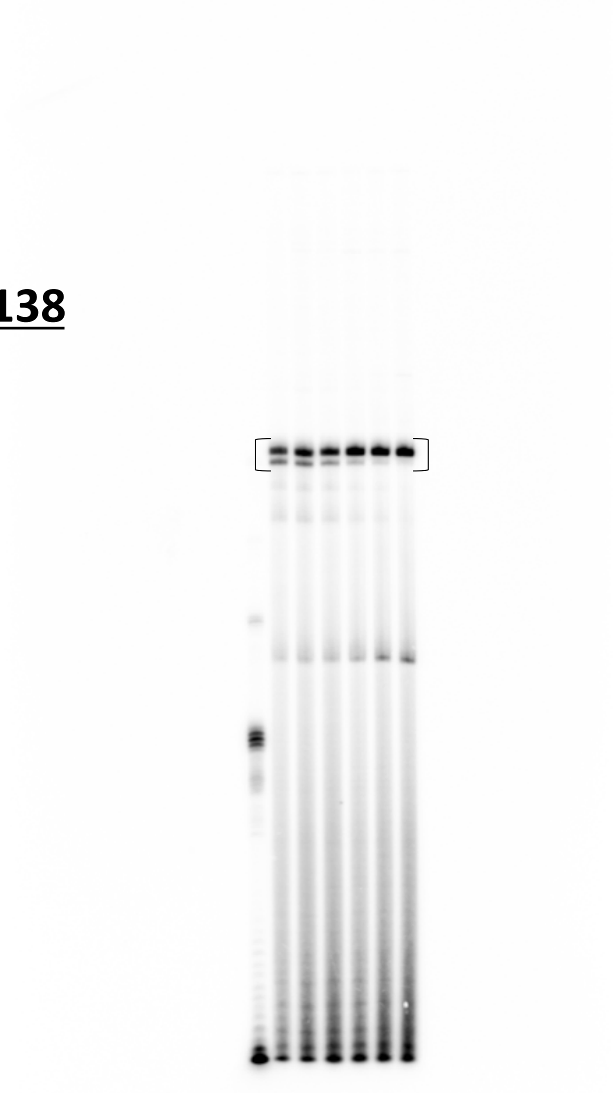

Fig 3B: AU-rich

- 20℃
- 24℃
- 28℃
- 32℃
- 36℃
- 40℃

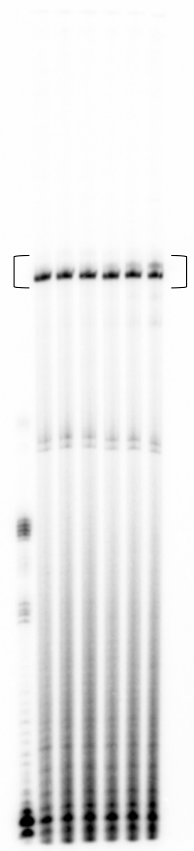

Fig 3B: Mut-IBS2

- 20℃
- 24℃
- 28℃
- 32℃
- 36℃
- 40℃

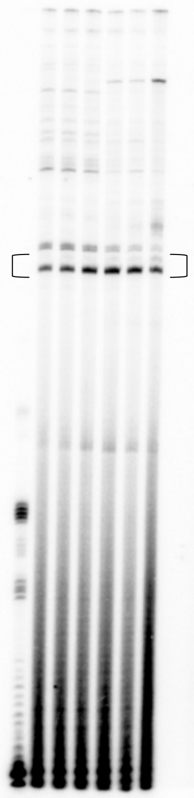

Fig 3B: IBS1-EBS1 swap

- 20℃
- 24℃
- 28℃
- 32℃
- 36℃
- 40℃

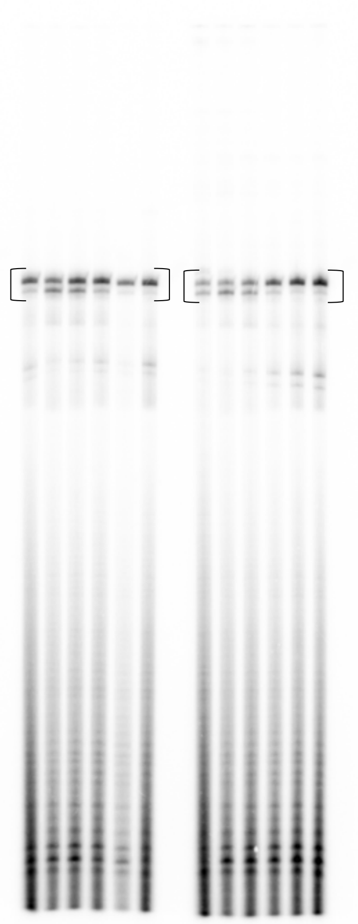

Fig 3B: IBS2-EBS2 swap

- 20℃
- 24℃
- 28℃
- 32℃
- 36℃
- 40℃

Fig 4A-B

- Ctl PCR
- Ll.LtrB
- Ef.PcfG
- Ll.RlxA
- Empty lane
- Ll.LtrB
- EF.PcfG
- Ll.RlxA
- Ll.LtrBΔA

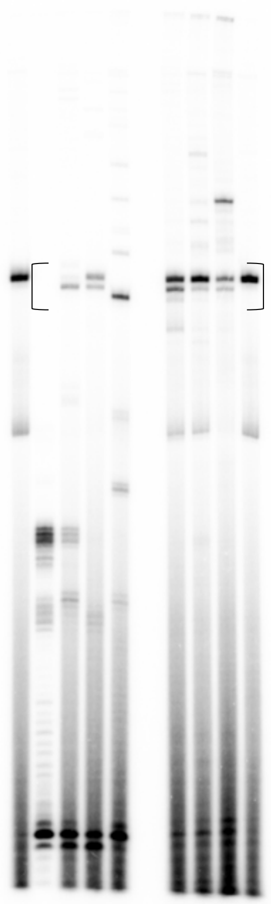

Fig 4C-D

- Ctl PCR
- Ll.LtrB
- Ef.PcfG
- Ll.RlxA
- Ll.LtrBΔA
- Ll.LtrB
- EF.PcfG
- Ll.RlxA
- Ll.LtrBΔA

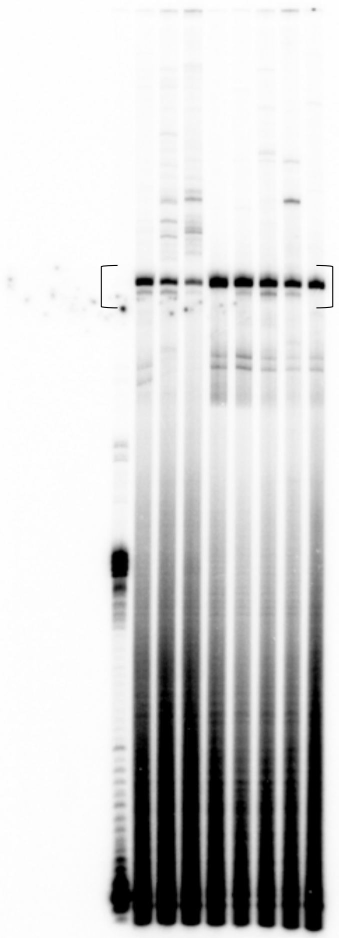

Fig 5

- Ctl PCR
- Ll.LtrB
- Ef.PcfG
- Ll.RlxA
- Ll.LtrB
- EF.PcfG
- Ll.RlxA
- Ll.LtrB
- Ef.PcfG
- Ll.RlxA
- Ll.LtrB
- Ef.PcfG
- Ll.RlxA

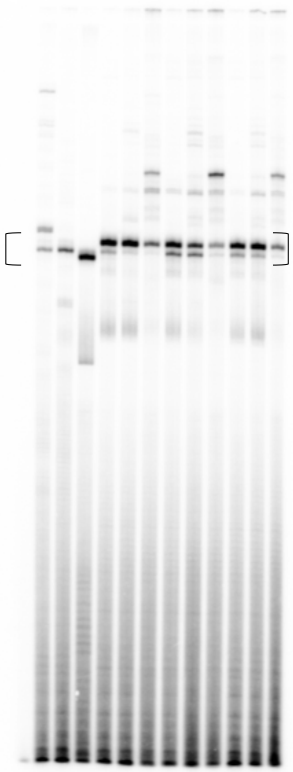

Supplement: S1 Raw images — (PDF) [file pone.0237367.s001.pdf]
